# Rearing substrate impacts growth and macronutrient composition of *Hermetia illucens* (L.) (Diptera: Stratiomyidae) larvae produced at an industrial scale

**DOI:** 10.1038/s41598-020-76571-8

**Published:** 2020-11-10

**Authors:** Andrea Scala, Jonathan A. Cammack, Rosanna Salvia, Carmen Scieuzo, Antonio Franco, Sabino A. Bufo, Jeffery K. Tomberlin, Patrizia Falabella

**Affiliations:** 1 Department of Science, University of Basilicata, Potenza, Italy; 2 Department of Entomology, Texas A&M University, College Station, TX USA; 3 Department of Geography, Environmental Management & Energy Studies, University of Johannesburg, Johannesburg, South Africa

**Keywords:** Environmental economics, Entomology

## Abstract

Organic waste is a rapidly increasing problem due to the growth of the agricultural production needed to meet global food demands. Development of sustainable waste management solutions is essential. Black soldier fly, *Hermetia illucens* (L.) (Diptera: Stratiomyidae) (BSF), larvae are voracious consumers of a wide range of organic materials ranging from fruits and vegetables to animal remains, and manure. Thanks to this ability and considering the larval high protein and lipid content, BSF larvae are a useful additive in animal feeds and biodiesel production. Unfortunately, the feasibility of using the black soldier fly as a tool for waste valorization and feed production has primarily been investigated at the benchtop scale. Thus, mobilization of current practices to an industrial scale is challenging because scaling up from small laboratory studies to large industrial studies is not necessarily linear. The goal of this study was to demonstrate the ability of the BSF to recycle organic waste at an industrial scale. To accomplish this goal, three organic waste streams were used (e.g., apples, bananas, and spent grain from a brewery) to test six diet treatments (1) apple, (2) banana, (3) spent grain, (4) apple and banana, (5) apple and spent grain, and (6) banana and spent grain. Working at scale of 10,000 BSF larvae life history traits, waste valorization, protein and lipid profiles were measured for each diet treatment. Differences were recorded across all variables, except substrate conversion, for larvae fed on fruit and spent grain (alone or with fruit). Growth rate significantly differed across treatments; larvae reared on spent grain grew twice as fast as those fed apples alone, but those reared on the apple and spent grain mixture produced twice as much insect biomass. However, it should be noted that larvae resulting from the apple diet contained 50% more fat than larvae fed the fruit and spent grain mixtures. Commonly-available organic wastes were successfully used at an industrial scale to produce BSF larvae that have the potential to substitute other sources of protein and lipids in different industrial applications. Industrialization efforts are encouraged to assess these impacts when integrating diverse ingredients into larval diets as a means to more precisely predict output, such as larval development time and final larval biomass.

## Introduction

Organic waste management is a rapidly growing, yet difficult problem to solve, given increased waste production and high costs of disposal^1^. The agricultural sector has grown considerably in the last two decades, both in land use and production, as a means to fulfill global demand for food^2^.

Among agri-food companies, breweries generate different by-products, such as spent grains and brewers’ yeast. These by-products are produced in huge amount daily^3,4^. Only a small amount of these by-products streams is used as livestock feed, as the shelf life of spent grain is less than 48 h. Most brewing by-products end up in landfills, which could pollute the surrounding environment^5^. Specifically, each year brewers produce about 38.6 million tons of spent grain^6^ and the main use for its valorisation is as animal feed due to its high protein and nutrition content^7^. Brewing has negative environmental impacts both during production and waste management, and spent grain constitutes the largest waste by volume, followed by brewers’ yeast. Almost 70% of spent grain is used as feed, but is a very wet product, with up to 80% water content and high microbial load. For this reason, in its raw form, spent grain needs to be re-used very quickly before its deterioration (around 48 h). Around 10% of spent grain is used to produce biogas, and the remaining 20% is landfilled. Every tonne of spent grain in a landfill releases 513 kg CO_2_ equivalent of greenhouse gases^8^.

Developing sustainable waste management methods for these agricultural by-products is essential. Not only are such methods needed to protect the environment, they could also serve as new economic opportunities. In the context of waste valorisation, the use of insects to recycle such wastes has opened up new frontiers in bioconversion strategies^9^. The ability of saprophagous macroinvertebrates to convert a wide range of decomposing organic substrates, such as vegetable matter, animal manure, and other organic refuse, could provide a sustainable method for waste recycling, while at the same time producing valuable secondary products such as animal feed, compost, and biofuels.

Insects have the ability to convert these organic wastes into insect biomass, with higher efficiency compared to poultry, cattle, and swine^10–12^. Moreover, rearing insects at an industrial scale emits lower greenhouse gases compared to conventional farming methods and requires less water and land^10,11,13^. Thus, rearing insects on agri-food waste for use as feed for livestock is potentially more sustainable and efficient than intensive cultivation of cereals and legumes intended for this purpose^10^.

The black soldier fly (BSF), *Hermetia illucens* (L.) (Diptera: Stratiomyidae), is a saprophytic insect, which currently has a near cosmopolitan distribution in the tropical and temperate areas^14^. During its larval stage, BSF larvae are voracious consumers of a wide variety of organic material, ranging from fruits and vegetables to animal remains, and manure^15,16^. Small-scale waste management using BSF larvae has already been tested with a variety of organic by-products, such as rice straw^17^, grains^18^, faecal sludge and manure^19,20^, and kitchen waste^16^. Harvested larvae are high in protein (41–44%) and lipid (15–49%) content, which can be used as animal feed^21–23^ and biodiesel production^17,24,25^; however, the use of the larvae is dependent on their nutritional makeup which is directly a result of what they feed on^26,27^.

The economic importance of BSF as a potential candidate for mass rearing is well established. But, knowledge on important aspects of BSF feeding performance on different agro-industrial waste streams at an industrial scale remains largely unknown. Historically, studies have been done at benchtop scale (i.e., several hundred larvae per replicate on grams of substrate) which may not truly represent what occurs on a larger industrial scale (thousand larvae fed on kilograms of diet).

The translation from benchtop to industrial scale is not necessarily linear, thus moving from benchtop results to industrial production, without first testing results at the industrial level, is not acceptable. Many studies are related to the possible modifications caused by scaling from laboratory to mass rearing^28,29^. For example, it was observed that mass rearing system positively influenced number of progeny (higher) and developmental time (shorter) of *Cylas formicarius elegantulus* (Coleoptera: Brentidae)^30^ and weight and size (both higher) of *Aedes aegypti* (Diptera: Culicidae) and *Aedes albopictus* (Diptera: Culicidae) eggs^31^. Moreover, higher density of individuals has potential advantages related to genetic variability in many species, including the ectoparasitoid *Habrobracon hebetor* (Hymenoptera, Braconidae)^32^ and *H. illucens* itself^33^. In particular, the high density of *H. hebetor* greatly improves genetic variability, fostering out-breeding and preventing side effects of inbreeding^32^ and the mass rearing of *H. illucens* (about 2500 adults for cage) counteract inbreeding depression^33^.

In this work, we fed BSF larvae on six types of agricultural by-products: Apple (A); Banana (B); Spent Grain from a local brewery (SG); Apple and Banana (AB) mixed in 1:1 ratio (w/w—weight per weight); Apple and Spent Grain (ASG) mixed in 1:1 ratio (w/w); Banana and Spent Grain (BSG) mixed in 1:1 ratio (w/w) and we examined larval development, growth, final biomass, protein and lipid content, and substrate reduction on an industrial scale. These waste streams are produced at a large scale, but everything we currently know about waste remediation using BSF is on a small scale; thus, we verified the need to test results on a larger scale.

The purpose of this study was to evaluate growth rate, total biomass and macronutrients of BSF fed commonly found agricultural by-products, whose availability is not linked to seasonality, by using industrial scale methods. Most of the data available on BSF growing performance and macronutrients composition originates from benchtop studies, which may not translate to a larger production scale. Results from this study provide a basis to compare findings from benchtop studies and help to optimize the BSF industrial rearing on vegetable waste streams.

## Results

### Larval growth rate

**Figure 1:**
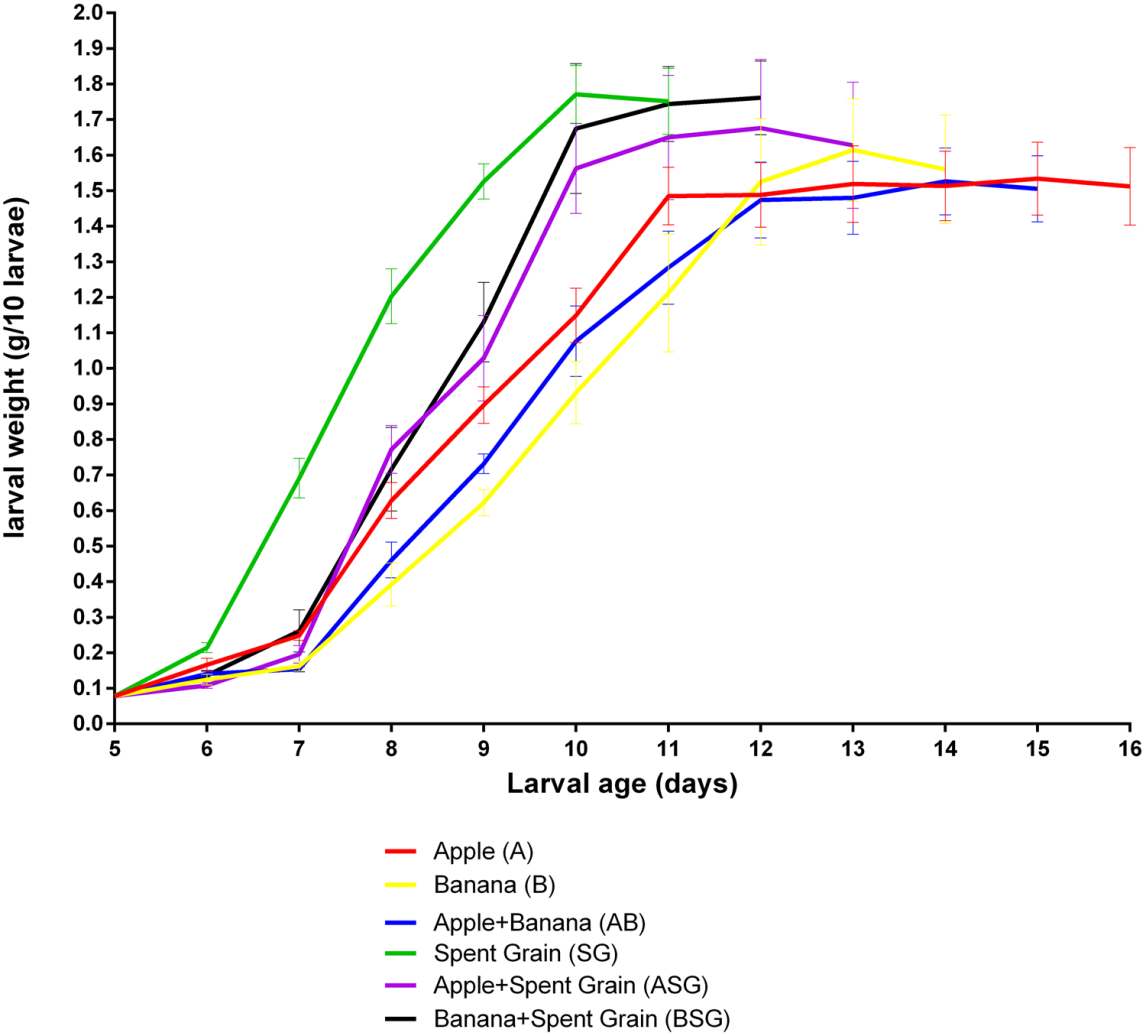
Daily average weight (mean ± SEM) (g/10 larvae) of BSF larvae reared on the six different diets; apple (A), banana (B), apple and banana mixed in 1:1 ratio (w/w—weight per weight) (AB), spent grain (SG), apple and spent grain mixed in 1:1 ratio (w/w) (ASG), banana and spent grain mixed in 1:1 ratio (w/w) (BSG).

BSF larval development on the experimental diets was compared from day 5 to day 11 (Fig. 1). Diet significantly affected larval growth (F_(5,30)_ = 12.22; p < 0.001). Differences among growth patterns depend on development time indicating that the pattern of the growth curves was significantly different among the experimental groups (F_(25,150)_ = 6.16; p < 0.001) (Fig. 1). Polynomial contrasts showed that the linear (F_(5,30)_ = 5.14; p = 0.002), quadratic (F_(5,30)_ = 16.16; p < 0.001) and cubic (F_(5,30)_ = 4.69; p = 0.03) components accounted for the differences among the shape of the growth curves. Polynomial tests of higher orders showed no differences.

We evaluated both the larval growth and the time length of larval development with an index of growth by time. This index returns the evaluation of the “best diet”, that is the one on which BSF larvae can convert the higher biomass in the shorter time. This index was different in a highly significant way (F_(5,30)_ = 19.43; p < 0.001) among the experimental groups. Any diet with spent grain (either alone or in combination with fruit) returned the highest value of this index, while all the other diets scored the lowest values.

### Diet reduction

Diet type significantly impacted both the reduction of diets by BSF and the amount of residue remaining (combination of left-over diet and frass), where diet reduction ranged from 59 to 74% and residue remaining ranged from 2.0 to 3.0 kg (F_(5,30)_ = 3.011; p = 0.0256) (Table 1). However, differences were only found between the AB and ASG diet treatments, while all other diet treatments showed similar rates of diet reduction and residue remaining.

**Table 1:** Index of growth by time (g/d), diet reduction (%), larvae total biomass (g), residue total weight (g), crude protein (%) and crude lipid (%) content of BSF larvae fed on the six different diets.

Diets	Index of growth by time (g/d)	Diet reduction (%)	Larvae total biomass (g)	Residue total weight (g)	Crude protein content (%)	Crude lipid content (%)
A	55.13 ± 2.9^a^	64.4 ± 4.8^ab^	882.1 ± 47.7^a^	2740.3 ± 371.7^ab^	31.12 ± 1.54^a^	36.1 ± 1.5^b^
B	75.18 ± 3.0^a^	63.4 ± 3.3^ab^	1052.5 ± 42.0^ab^	2811.6 ± 255.4^ab^	36.50 ± 0.88^b^	27.9 ± 3.9^ab^
AB	69.59 ± 2.5^a^	59.4 ± 1.7^a^	1043.8 ± 38.1^ab^	3121.6 ± 136.3^b^	35.60 ± 1.19^ab^	33.4 ± 1.8^ab^
SG	124.06 ± 10.8^b^	68.5 ± 1.4^ab^	1364.6 ± 118.7^bc^	2419.3 ± 111.8^ab^	47.83 ± 1.04^c^	22.5 ± 1.5^a^
ASG	127.15 ± 11.1^b^	74.0 ± 2.6^b^	1653.0 ± 144.9^c^	2001.5 ± 203.2^a^	48.01 ± 1.5^c^	20.1 ± 4.3^a^
BSG	121.08 ± 7.4^b^	69.7 ± 2.6^ab^	1453.0 ± 88.9^c^	2329.0 ± 203.1^ab^	45.61 ± 0.50^c^	23.1 ± 2.3^a^

Diet moisture content (%) was calculated on 10 g of substrates, dried for 24 h at 55 °C, as the difference between the weight before and after drying using an analytical balance. Ten larvae were randomly sampled with replacement daily from each replicate and weighed. The end of the experiment for each tested diet was determined when, following daily measures, no increase or a decrease in larval weight was recorded. The index of growth by time was generated applying Eq. (1). Percentage of substrate reduction (on a wet basis) was calculated applying Eq. (2). Total biomass (g) of BSF larvae and total weight of residue (g) derived from BSF larvae were measured at the end of the experiment, separating BSF larvae from residue. Larvae and residue were weighed separately. Twenty grams of larvae were harvested from each diet and used for crude protein and lipid analyses (i.e. 10 g per analysis), on a dry basis. The Kjeldahl method was used for crude protein content analysis. For crude lipid content larvae were first dried and then immersed in chloroform to extract the fat body. The residual dry matter was used to calculate the percentage of lipids in each group of larvae fed with the different diets applying Eq. (3). Data are presented as means ± SEM (standard error of the mean) and compared by One-Way Analysis of Variance (ANOVA) and Bonferroni *post-hoc* test. Different letters indicate a significant difference (p < 0.05). A = apple; B = banana; AB = apple and banana mixed in 1:1 ratio (w/w—weight per weight); SG = spent grain; ASG = apple and spent grain mixed in 1:1 ratio (w/w); BSG = banana and spent grain mixed in 1:1 ratio (w/w).

### Larvae total biomass

Diet significantly affected total larval biomass (F_(5,30)_ = 10.71; df 5, 30; p < 0.0001). Larvae reared on diets containing spent grain (ASG and BSG) were significantly higher in biomass (1.5 kg on average) than those on fruit alone (A, B and AB) (1.0 kg on average) (Table 1).

### Crude protein and lipid content

Diet significantly affected crude protein content of the larvae (F_(5,30)_ = 38.59; p < 0.0001). Larvae fed on diets containing spent grain (SG, ASG, BSG) contained 15% or more protein than those reared on fruit only diets (Table 1). We observed that larvae fed on diet A also differed from larvae fed on diet containing B (Table 1). Diet significantly affected also crude lipid content (F_(5,30)_ = 5.414; p = 0.0012). We observed that larvae fed on A diet, with on average 36% of lipids, differed from larvae fed on all diets containing SG, characterized by about 22% of lipids, but did not differ from larvae fed on diets without SG (on average 30.65%) (Table 1).

## Discussion

BSF growth and biomass at the end of the bioconversion process, as well as nutrient composition in terms of crude protein and lipid content, were dependent on the provided diet and the rearing conditions. To the best of our knowledge, this is the first time that the real feasibility of organic waste bioconversion process mediated by BSF is shown using industrial quantities of commonly-found agricultural by-products. Historically most studies operate at a benchtop or laboratory scale, in which larval numbers and substrate amount are reflective of these smaller scale studies, which may impact BSF development. This limitation (i.e., lack of industrial scale data) could prove problematic for future applications as data most likely do not scale linearly. This concept is not new to science: the transition from laboratory to mass rearing, in fact, could bring about variations in life history parameters^28,29,34^. Also *H. illucens* mobilization from benchtop to the industrial scale could produce significant change, could be not linear, as in this context, and different factors could influence BSF production and performances.

Many examples of BSF reared at laboratory scale on fruits and on spent grain are reported^33,35–40^ (Supplementary Tables 1, 2). When working on a laboratory scale, with small quantities of larvae, BSF performances clearly vary based on the rearing conditions (substrate, temperature and relative humidity), even in a relevant way regarding time to reach the prepupal instar and the recorded prepupal weight (Supplementary Tables 1, 2).

When BSF benchtop and mass rearing are compared in similar conditions (substrate, temperature and relative humidity), differences in developmental time and final prepupal weight are recorded. In benchtop studies developmental time is longer and single prepupal weight is really lower compared to our findings: in laboratory studies larvae take from 6 to 24 extra days to reach the prepupal stage (Supplementary Tables 1, 2); moreover, the prepupal instar is reached with a weight of 2 to 50-times lower (Supplementary Tables 1, 2). Exceptions are found in Nguyen et al.^37^ and in Chia et al.^33^, in which BSF larvae fed on fruit and vegetable mix and spent grain respectively, despite their longer development time, have similar weight to prepupal fed on mass rearing condition, and in Meneguz et al.^40^, in which larvae fed on spent grain, although the lower weight, reach the prepupal stage in brief time, more similar to our findings.

Beside the environmental temperature, the large amount of BSF larvae reared in an industrial process surely generates heat^41^, that, consequently, positively influences BSF development performances. In literature, there are many examples of larvae reared individually or in small groups growing more slowly than larvae reared in large groups^42–44^. The external heat can also influence the regulation of larval gut enzymes, inducing a faster proteolytic activity in BSF midgut, which optimum is about 45 °C^45^.

Similar to the laboratory scale experiments, also in our work BSF larvae fed on fruit have the longest development time and the lower biomass compared to spent grain substrate, probably due to the low protein content of these substrates (A: 0.26 g/100 g; B: 1.09 g/100 g, according to data extrapolated from USDA Food Composition Databases)^11,35,46,47^. Furthermore, A, B, and AB have higher moisture content in comparison to diets containing spent grain, resulting in a lower quantity of nutrients considering their equal weight. Combined protein and moisture content likely influenced larval development on these substrates^11,47^. BSF larvae are able to grow on diets with low protein content, although with a prolonged time of feeding to reach their final weight, compared to larvae fed on diets richer in proteins^35,40^. The high fibre content of apples and bananas did not appear to impact the ability of larvae to digest these diets, which could be in part due to gut bacteria able to digest cellulose^48^. The fast growth rate and high larval biomass observed in SG larvae and, above all, on diets containing SG mixed with fruit, could be attributed to a more balanced ratio between proteins and carbohydrates^26,35^. Apple and banana, in fact, are substrates rich in carbohydrates (A: 13.81 g/100 g; B: 22.84 g/100 g, source: USDA Food Composition Databases) and BSF are able to convert these carbohydrates into lipids^49,50^, the energy storage molecules later used by the adult flies^51^. Larvae reared on energy-dense diets produce prepupae with a high fat content, rich in medium chain fatty acids, that could provide an added value in comparison to conventional feed resources^49^.

Summarizing, moving from small to larger scale the expected linear increase in biomass is not observed, but surprisingly a consistent improvement of BSF performance efficiency is recorded, both in prepupal instar development time (lower) and prepupal weight (higher). The quickness of mass rearing process could have positive implications in waste disposal and in rearing costs: benchtop conditions could negatively affect the development and the digestion processes in BSF larvae, compromising their growth; on the contrary, mass rearing, with a shorter development time and a greater final larval biomass reduce costs of rearing and allows the achievement of much more product of high economic and biological value (protein- and fat-rich larvae). Results obtained in our study and the comparison with other small scale studies show that scale conditions influence the BSF rearing and that the transition from a benchtop to a large/industrial scale may not be linear for all life history traits and valorization variables (e.g. waste reduction). Applications of benchtop data could result in under, or even over, estimations of expected output, in fact growth, development and final biomass of BSF are higher for insects reared at industrial scale compared to benchtop or even individually rearing. Such miscalculations could have significant consequences when attempting to build a facility and predict output. Future research should incorporate industrialized versions of treatments along with benchtop as a mean to allow more accurate interpretations. At minimum, this limitation should be presented when conducting benchtop studies. However, the same can be said when drawing generalized conclusions without considering matters such as, but not limited to, experimental design and population genetics, outcomes are specific^52–54^. The mass rearing condition reproduces more accurately BSF natural attitude of developing in large aggregate masses and gregariously feeding on their substrates^55^. For waste management, large scale studies are ideal and strictly necessary, since no linear correlation with laboratory studies are detected. Benchtop studies, on the other hand, could be useful to firstly verify with a more quick and economic method different substrates on which *H. illucens* could be reared on and progressively move to the industrial scale (scaling up).

In this study, we demonstrated that commonly available organic waste of the agri-industrial sector such as apple, banana and spent grain can be successfully used at an industrial scale to produce high quality BSF larvae that have the potential to substitute other sources of protein and lipids in commercial livestock feed and energy production, respectively. Specific diets or mixes of them should be selected in order to produce insects with the desired nutrient profile to satisfy the needs of the intended markets. SG, ASG and BSG larvae were the most successful groups considering the total larval biomass and crude protein content. BSF fed on these kinds of diets are good candidates for the production of high protein flours and protein-enriched meals, representing a good feed alternative both to the conventional fish meal and above all to proteins of vegetable origin (e.g. soy). Moreover, this study could be helpful to optimize the industrial scale application of *H. illucens* feeding, which would greatly reduce the ecological and economic footprint of feed and energy production, thereby contributing to more sustainable productive systems.

## Methods

### Insect rearing

Black soldier fly eggs were collected from a colony maintained in the Forensic Laboratory for Investigative Entomological Sciences (F.L.I.E.S. Facility) at Texas A&M University (College Station, TX, USA) in 5(L) × 3(W) × 3(H) cm triple-layer corrugated cardboard egg traps^56^ and stored in 500 ml glass jars in an environmental chamber (Rheem Environmental Chamber) at 27.0 ± 1.0 °C, 70.0% relative humidity (RH), 14:10 L:D until hatching. After hatching, approximately 60,000 neonate larvae were divided among six diets (10,000 per diet) and placed in 500 ml plastic containers. They were provided 200 g of Gainesville diet (30% alfalfa, 50% wheat bran, 20% corn meal)^57^ at 70% moisture for four days. Containers of larvae were kept in the same environmental chamber at 27.0 ± 1.0 °C, 70.0% relative humidity (RH). We replicated this study six times.

### Experiment trials

Five-day old larvae including all the contents of the 500 ml plastic containers (larvae, residue, dry Gainesville diet) were transferred to Sterilite^®^ plastic trays (56 cm × 40 cm × 13 cm) containing 7.0 kg of their respective diet treatment (1) Apple (A); (2) Banana (B); (3) Spent Grain from brewery waste (SG); (4) Apple and Banana (AB) mixed in 1:1 ratio (w/w—weight per weight); (5) Apple and Spent Grain (ASG) mixed in 1:1 ratio (w/w); (6) Banana and Spent Grain (BSG) mixed in 1:1 ratio (w/w). Dry Gainesville diet was placed around the perimeter of diets to prevent larvae from escaping. Plastic trays with larvae were placed in the previously described environmental chamber. Fruit was purchased from a local grocery store (Wal-Mart Supercenter, College Station, TX, USA) and used fresh; spent grain was provided by a local brewery. Apples and bananas (whole fruit with peels and cores) were cut into small pieces using a fruit crusher (50 cm × 31 cm × 26 cm) (Model 1141 EJWOX, Santa Ana, CA, USA) prior to the use. Moisture content of the six diets was calculated on 10 g of substrates, dried for 24 h at 55 °C in a Precision Scientific Thelco Oven (Thermo Fisher Scientific, Waltham, MA, USA), as the difference between the weight before and after drying using an analytical balance (Adventure Pro balance, Ohaus, Pine Brook NJ, USA) (Supplementary Figure 1). Six replicates were completed over two experimental periods (i.e. three replicates per trial).

### Growth rate, larval biomass and diet reduction

To quantify larval growth rate and biomass, larvae were sampled daily to record their weight. Starting with 5-day-old larvae, samples (with replacement) of ten larvae were collected and weighed on an analytical balance (Adventure Pro balance, Ohaus, Pine Brook NJ, USA). Sampling continued within a diet treatment until the first day of larval weight decreasing (prepupal instar).

An index of growth by time was generated for each diet treatment applying the following Eq. (1):

1 $$\text{Index of growth by time}=\frac{\text{Total larval biomass }\left(\text{g}\right)}{\text{Days of experimental trial }\left(\text{d}\right)}$$

When larval weight decreased, BSF larvae were separated from their diets and collected manually after sieving the residue, a combination of left-over diet (not consumed) and frass. Larvae and residue were weighed separately. Diet reduction was calculated applying the following Eq. (2)^29^:

2 $$\text{Diet Reduction }(\text{\%})=\frac{\text{Initial diet }\left(\text{g}\right)-\text{Residue }(\text{g})}{\text{Initial diet }(\text{g})} * 100$$

### Crude protein and lipid analysis

Crude proteins and lipids were analysed at the end of the experiment. Twenty grams of larvae were harvested from each diet and used for crude protein and lipid analyses (i.e. 10 g per analysis). Larvae were first frozen at − 20 °C and then sent to SDK Laboratories, Inc., Hutchinson, KS, USA, where they were used for crude protein analysis on a dry basis using the Kjeldahl method. For lipid analysis we followed modified methods reported by Loveridge^58^. Larvae were dried for 24 h at 55 °C in a Precision Scientific Thelco Oven (Thermo Fisher Scientific, Waltham, MA, USA) to remove water. Then they were weighed on an Adventurer Pro balance (Ohaus, Pine Brook, NJ) (initial dry weight). To extract the fat body, they were immersed in three changes (24 h each) of cold chloroform in glass test tubes. The first two changes of chloroform became yellowish, while the last remained colourless indicating that the fat extraction was complete. Larvae were then dried for 2 h in the aforementioned oven and reweighed. This second weighing gave the dry mass deprived by chloroform-soluble substances (final dry weight). This residual dry matter was used to calculate the percentage of lipids in each group of larvae fed with the different diets. The Eq. (3) was applied:

3 $$\text{Crude lipids }(\text{\%})=\frac{\text{Larvae initial dry weight }\left(\text{g}\right)-\text{Larvae final dry weight }(\text{g})}{\text{Larvae initial dry weight }(\text{g})} * 100$$

### Statistical analysis

Growth curves of the larval biomass of each group fed on the different diets were compared by Repeated Measures Analysis of Variance^59^. The interaction of diet and the within-factor time was tested using linear, quadratic and higher-order polynomial contrasts in order to assess differences in the slope of the growth curves. Compound symmetry was checked through Huynh–Feldt statistics (Systat 13, Systat Software Inc.).

Crude protein and lipid content, total larval biomass, diet moisture, residue remaining (combination of left-over diet and frass), index of growth by time, and percentage of diet reduction were analyzed by One-Way Analysis of Variance (ANOVA) and Bonferroni *post-hoc* test. Normality of data was checked by Lilliefors corrected Kolmogorov–Smirnov test and Shapiro–Wilk test. Statistical analysis was performed with the statistical package Systat 13 (Systat Software Inc.).

For each analysis alpha was set at 0.05 for significance.

## Supplementary information

Supplementary Information.

## Data Availability

The datasets generated during the current study are available from the corresponding authors on request.

## References

[1] Otles S, Despoudi S, Bucatariu C, Kartal C, Galanakis CM (2015). Food waste management, valorization, and sustainability in the food industry. Food Waste Recovery.

[2] Schieber A, Stintzing FC, Carle R (2001). By-products of plant food processing as a source of functional compounds—Recent developments. Trends Food Sci. Technol..

[3] Gowe C (2015). Review on potential use of fruit and vegetables by-products as a valuable source of natural food additives. Food Sci. Qual. Manag..

[4] Chia SY (2018). Effects of waste stream combinations from brewing industry on performance of Black soldier fly, *Hermetia illucens* (Diptera: Stratiomyidae). PeerJ..

[5] Newman P, Jennings I (2008). Cities as Sustainable Ecosystems: Principles and Practices.

[6] Lynch KM, Steffen EJ, Arendt EK (2016). Brewers’ spent grain: A review with an emphasis on food and health. J. Inst. Brew..

[7] Bolwig, S., Mark, M. S., Happel, M. K. & Brekke, A. Beyond animal feed?: the valorisation of brewers’ spent grain. In *From Waste to Value: Valorisation Pathways for Organic Waste Streams in Circular Bioeconomies *(ed. Taylor & Francis) 107–126 (2019).

[8] Malakhova DV, Egorova MA, Prokudina LI, Netrusov AI, Tsavkelova EA (2015). The biotransformation of brewer’s spent grain into biogas by anaerobic microbial communities. World J. Microbiol. Biotechnol..

[9] Čičková H, Newton GL, Lacy RC, Kozánek M (2015). The use of fly larvae for organic waste treatment. Waste Manag..

[10] Van Huis A (2013). Potential of insects as food and feed in assuring food security. Ann. Rev. Entomol..

[11] Oonincx DGAB, Van Broekhoven S, Van Huis A, van Loon JJA (2015). Feed conversion, survival and development, and composition of four insect species on diets composed of food by-products. PLoS ONE.

[12] Wang YS, Shelomi M (2017). Review of black soldier fly (*Hermetia illucens*) as animal feed and human food. Foods..

[13] Costa-Neto EM (2013). Insects as human food: An overview. Amazon. Rev. Antropol..

[14] Diener, S. *et al*. Black soldier fly larvae for organic waste treatment–prospects and constraints. In *Proceedings, WasteSafe 2011—2nd Int. Conf. on Solid Waste Management in the Developing Countries* (eds. Alamgir, M. *et al.*) 52–59 (2011).

[15] Zhou F, Tomberlin JK, Zheng L, Yu Z, Zhang J (2013). Developmental and waste reduction plasticity of three black soldier fly strains (Diptera: Stratiomyidae) raised on different livestock manures. J. Med. Entomol..

[16] Nguyen T, Tomberlin JK, Vanlaerhoven S (2015). Ability of black soldier fly (Diptera: Stratiomyidae) larvae to recycle food waste. Environ. Entomol..

[17] Zheng L, Li Q, Zhang J, Yu Z (2012). Double the biodiesel yield: Rearing black soldier fly larvae, *Hermetia illucens*, on solid residual fraction of restaurant waste after grease extraction for biodiesel production. Renew. Energy..

[18] Webster CD (2016). Bio-ag reutilization of distiller’s dried grains with solubles (DDGS) as a substrate for black soldier fly larvae, *Hermetia illucens*, along with poultry by-product meal and soybean meal, as total replacement of fish meal in diets for Nile tilapia, *Oreochromis niloticus*.. Aquacult. Nutr..

[19] Lalander C (2013). Faecal sludge management with the larvae of the black soldier fly (*Hermetia illucens*)—From a hygiene aspect. Sci. Total Environ..

[20] Banks IJ, Gibson WT, Cameron MM (2014). Growth rates of black soldier fly larvae fed on fresh human faeces and their implication for improving sanitation. Trop. Med. Int. Health..

[21] Barroso FG (2014). The potential of various insect species for use as food for fish. Aquaculture.

[22] Henry M, Gasco L, Piccolo G, Fountoulaki E (2015). Review on the use of insects in the diet of farmed fish: Past and future. Anim. Feed Sci. Technol..

[23] Surendra KC, Olivier R, Tomberlin JK, Rajesh Jha R, Khanalet SK (2016). Bioconversion of organic wastes into biodiesel and animal feed via insect farming. Renew. Energy..

[24] Leong SY, Kutty SRM, Malakahmad A, Tan CK (2016). Feasibility study of biodiesel production using lipids of *Hermetia illucens* larva fed with organic waste. Waste Manag..

[25] Li W (2015). Potential biodiesel and biogas production from corncob by anaerobic fermentation and black soldier fly. Bioresour Technol..

[26] Cammack JA, Tomberlin JK (2017). The impact of diet protein and carbohydrate on select life-history traits of the black soldier fly *Hermetia illucens* (L.) (Diptera: Stratiomyidae). Insects..

[27] Li W (2015). Simultaneous utilization of glucose and xylose for lipid accumulation in black soldier fly. Biotechnol. Biofuels..

[28] Soma DD (2017). Does mosquito mass-rearing produce an inferior mosquito?. Malar. J..

[29] Sørensen J, Addison M, Terblanche J (2012). Mass-rearing of insects for pest management: Challenges, synergies and advances from evolutionary physiology. Crop Prot..

[30] Kuriwada T, Kumano N, Shiromoto K, Haraguchi D (2010). Effect of mass rearing on life history traits and inbreeding depression in the sweetpotato weevil (Coleoptera: Brentidae). J. econ. entomol..

[31] Zheng ML, Zhang DJ, Damiens DD, Yamada H, Gilles JR (2015). Standard operating procedures for standardized mass rearing of the dengue and chikungunya vectors *Aedes aegypti* and *Aedes albopictus* (Diptera: Culicidae)—I—egg quantification. Parasit. Vectors..

[32] Ghimire MN, Phillips TW (2010). Mass rearing of *Habrobracon hebetor* Say (Hymenoptera: Braconidae) on larvae of the Indian meal moth, *Plodia interpunctella* (Lepidoptera: Pyralidae): Effects of host density, parasitoid density, and rearing containers. J. Stored Prod. Res..

[33] Chia SY (2018). Threshold temperatures and thermal requirements of black soldier fly *Hermetia illucens*: Implications for mass production. PLoS ONE.

[34] McGill BJ (2010). Matters of scale. Science.

[35] Jucker C, Erba D, Leonardi MG, Lupi D, Savoldelli S (2017). Assessment of vegetable and fruit substrates as potential rearing media for *Hermetia illucens* (Diptera: Stratiomyidae) larvae. Environ. Entomol..

[36] Jucker C, Leonardi MG, Rigamonti I, Lupi D, Savoldelli S (2020). Brewery’s waste streams as a valuable substrate for Black Soldier Fly *Hermetia illucens* (Diptera: Stratiomyidae). J. Entomol. Acarol. Res..

[37] Nguyen TTX, Tomberlin JK, Vanlaerhoven S (2013). Influence of resources on *Hermetia illucens* (Diptera: Stratiomyidae) larval development. J. Med. Entomol..

[38] Barbi S (2020). Valorization of seasonal agri-food leftovers through insects. Sci. Total Environ..

[39] Bava L (2019). Rearing of *Hermetia illucens* on different organic by-products: Influence on growth, waste reduction, and environmental impact. Animals.

[40] Meneguz M (2018). Effect of rearing substrate on growth performance, waste reduction efficiency and chemical composition of black soldier fly (*Hermetia illucens*) larvae. J. Sci. Food Agric..

[41] Slone D, Gruner SV (2007). Thermoregulation in larval aggregations of carrion-feeding blow flies (Diptera: Calliphoridae). J. Med. Entomol..

[42] Gere G (1956). Investigations into the laws governing the growth of *Hyphantria cunea* drury caterpillars. Acta Biol. Hung..

[43] Long DB (1953). Effects of population density on larvae of Lepidoptera. Trans. R. Entomol. Soc. Lond..

[44] Parra Paz AS, Carrejo NS, Gómez Rodríguez CH (2015). Effects of larval density and feeding rates on the bioconversion of vegetable waste using black soldier fly larvae *Hermetia illucens* (L.), (Diptera: Stratiomyidae). Waste Biomass Valor..

[45] Bonelli M (2019). Structural and functional characterization of *Hermetia illucens* larval midgut. Front. Physiol..

[46] Tschirner M, Simon A (2015). Influence of different growing substrates and processing on the nutrient composition of black soldier fly larvae destined for animal feed. J. Insect Food Feed..

[47] Gobbi P, Martinez Sanchez A, Rojo S (2013). The effects of larval diet on adult life history traits of the black soldier fly, *Hermetia illucens* [Diptera: Stratiomyidae]. Eur. J. Entomol..

[48] Kim E, Park J, Lee S, Kim Y (2014). Identification and physiological characters of intestinal bacteria of the black soldier fly, *Hermetia illucens*. Korean J. Appl. Entomol..

[49] Spranghers T (2017). Nutritional composition of black soldier fly (*Hermetia illucens*) prepupae reared on different organic waste substrates. J. Sci. Food Agric..

[50] Inagaki S, Yamashita O (1986). Metabolic shift from lipogenesis to glycogenesis in the last instar larval fat body of the silkworm, *Bombyx mori*. Insect Biochem..

[51] Tomberlin JK, Sheppard DC, Joyce JA (2002). Selected life-history traits of black soldier flies (Diptera: Stratiomyidae) reared on three artificial diets. Ann. Entomol. Soc. Am..

[52] Bosch G (2020). Standardisation of quantitative resource conversion studies with black soldier fly larvae. J. Insects Food Feed..

[53] Ståhls G (2020). The puzzling mitochondrial phylogeography of the black soldier fly (*Hermetia illucens*), the commercially most important insect protein species. BMC Evol. Biol..

[54] Zhan S (2019). Genomic landscape and genetic manipulation of the black soldier fly *Hermetia illucens*, a natural waste recycler. Cell. Res..

[55] Barragan-Fonseca KB, Dicke M, van Loon JJA (2017). Nutritional value of the black soldier fly (*Hermetia illucens* L.) and its suitability as animal feed—A review. J. Insects Food Feed.

[56] Booth DC, Sheppard C (1984). Oviposition of the black soldier fly, *Hermetia Illucens* (Diptera: Stratiomyidae): Eggs, masses, timing, and site characteristics. Environ. Entomol..

[57] Hogsette JA (1992). New diets for production of house flies and stable flies (Diptera: Muscidae) in the laboratory. J. Econ. Entomol..

[58] Loveridge JP (1973). Age and the changes in water and fat content of adult laboratory- reared *Locusta migratoria migratorioides*. Rhod. J. Agric. Res..

[59] Sokal RR, Rohlf FJ (1995). Biometry: The Principles and Practice of Statistics in Biological Research.

