# Supplementary material for: Rearing substrate impacts growth and macronutrient composition of Hermetia illucens (L.) (Diptera: Stratiomyidae) larvae produced at an industrial scale
Source: Sci Rep. 2020 Nov 10;10:19448. doi: 10.1038/s41598-020-76571-8 (PMC7655861; doi:10.1038/s41598-020-76571-8)
Supplement: Supplementary file 1 — Supplementary Information. [file 41598_2020_76571_MOESM1_ESM.docx]

**Supplementary Information**

**Rearing substrate impacts growth and macronutrient composition of *Hermetia illucens* (L.) (Diptera: Stratiomyidae) larvae produced at an industrial scale**

Andrea Scala^1^, Jonathan A. Cammack^2^, Rosanna Salvia^1^, Carmen Scieuzo^1^, Antonio Franco^1^, Sabino A. Bufo^1,3^, Jeffery K. Tomberlin^2*^ and Patrizia Falabella^1*^

*Corresponding authors:

- Jeffery K. Tomberlin, Department of Entomology, Texas A&M University, College Station, TX, USA

[jktomberlin@tamu.edu](mailto:jktomberlin@tamu.edu)

- Patrizia Falabella, Department of Science, University of Basilicata, Potenza, Italy

[patrizia.falabella@unibas.it](mailto:patrizia.falabella@unibas.it)


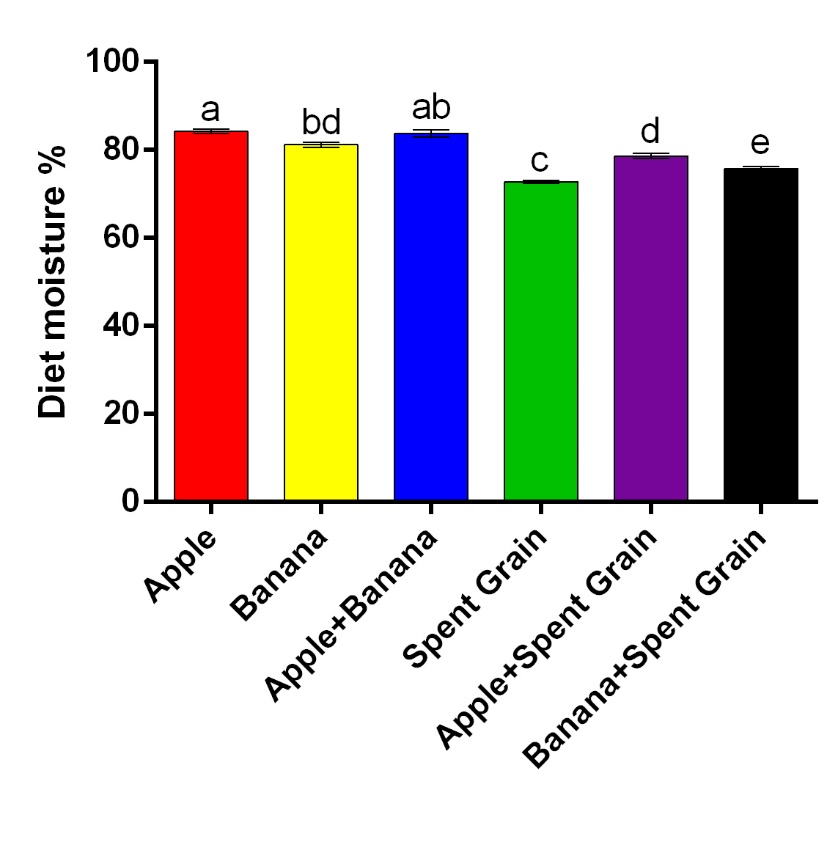


**Supplementary Figure 1:**

Moisture of the six diets was calculated at the beginning of the experiment. 10 g of each diet in six replicates was weighed, placed into an aluminium dish and dried for 24h at 55°C in a Precision Scientific Thelco Oven (Thermo Fisher Scientific, Waltham, MA, USA). Moisture content in percentage was determined as the difference between the weight before drying and the weight after drying using an Adventure Pro Balance (Ohaus, Pine Brook NJ, USA).

Means were compared by analysis of variance (ANOVA) and Bonferroni *post-hoc* test. Different letters indicate a significant difference (p<0.05).

The six different diets used in the experiment had significantly different moisture contents (F_(5,30)_ = 61.66; p<0.0001) at the beginning of the experiment. Diets containing only fruit had moisture contents above 80%. On average, A had the highest moisture content (84.2 ± 0.5%), followed by AB (83.8 ± 0.8%) and B (81.19 ± 0.5%). The other three substrates had lower moisture contents: ASG was 78.64 ± 0.5%, followed by BSG (75.78 ± 0.6%) and SG (72.75 ± 0.3%).

| **References** | **Substrate** | **Rearing temperature (°C)** | **Rearing humidity (%)** | **Instar of bioassay end** | **Number of larvae** | **Total diet administered (g)** | **Diet for each larva (g)** | **Developmental time (days)** | **Larval weight/n larvae (g)** | **Single larval weight (g)** |
| --- | --- | --- | --- | --- | --- | --- | --- | --- | --- | --- |
| Scala *et al.* | Apple  Banana  Apple+Banana | 27.0 ± 1.0 | 70 | Prepupal | 10000 | 7000 | 0.7 | 14-16 | 1.526/10 | 0.15 |
| Barbi *et al.^38^* | Apple | 27 ± 0.5 | 60/70 | Prepupal | 100 | 25 to 375 | from 2.5 to 3.75 g | 25.3 |  | 0.0665 |
| Meneguz *et al.^40^* | Fruit mix (apple, orange, apple leftover, strawberry, mandarin, pear, kiwi, banana) | 27 ± 0.5 | 70 ± 5 | Prepupal | 100 | 100  (+ 50 in case of need) | 1 or 1.5 | 28.2 ± 0.98 | 0.18/30 | 0.006 |
| Jucker *et al.^35^* | Fruit mix (apple, pear, orange) | 25 ± 0.5 | 60 ± 0.5 | Prepupal | 200 | *ad libitum* |  | 52 | 0.174 ± 0.009/10 | 0.017 |
| Nguyen *et al.^37^* | Fruit and vegetable mix | 28 | 65 ± 10 | Prepupal | 150 | 6 to 11 each day | 0.04 to 0.07 | from 21.67 ± 0.333 to 40.33 ± 1.542 | 0.369/3 | 0.123 |

Supplementary Table 1: industrial scale (Scala *et al.*) compared to laboratory scale experiments on *Hermetia illucens* larvae fed on fruit substrates. In detail substrate, rearing conditions (temperature (°C) and relative humidity (%)), instar of bioassay end, number of reared larvae, total diet (g) and diet administered for each larva (g), developmental time (days), recorded larval weight for a specific amount of larvae and (g) single larval weight (g) are reported.

| **References** | **Substrate** | **Rearing temperature (°C)** | **Rearing humidity (%)** | **Instar of bioassay end** | **Number of larvae** | **Total diet administered (g)** | **Diet for each larva (g)** | **Developmental time (days)** | **Larval weight/n larvae (g)** | **Single larval weight (g)** |
| --- | --- | --- | --- | --- | --- | --- | --- | --- | --- | --- |
| Scala *et al.* | Spent grain  Spent grain+apple  Spent grain+banana | 27.0 ± 1.0 | 70 | Prepupal | 10000 | 7000 | 0.7 | 11-13 | 1713.74/10 | 0.17 |
| Chia *et al.^33^* | Spent grain + barley | 28 ± 1 | 70 ± 2 | Prepupal | 300 | *ad libitum* |  | 26 |  | 0.15 |
| Meneguz *et al.^40^* | Barley brewers’ grains | 27 ± 0.5 | 70 ± 5 | Prepupal | 100 | 100  (+ 50 in case of need) | 1 or 1.5 | 14.0 ± 0.01 | 0.12/30 | 0.004 |
| Jucker *et al.^36^* | Brewer’s spent grain | 25 ± 0.5 | 60 ± 0.5 | Prepupal | 200 | *ad libitum* |  | 24.7±0.3 | 0.86 ± 0.0003/10 | 0.086 |
| Bava *et al.^39^* | Brewer’s grains | 25 ± 0.5 | 60 ± 0.5 | Prepupal | 1000 | *ad libitum* |  | 22 ± 0.58 | 0.98 ± 0.01/10 | 0.098 |

Supplementary Table 2: industrial scale (Scala *et al.*) compared to laboratory scale experiments on *Hermetia illucens* larvae fed on spent grain substrates. In detail substrate, rearing conditions (temperature (°C) and relative humidity (%)), instar of bioassay end, number of reared larvae, total diet (g) and diet administered for each larva (g), developmental time (days), recorded larval weight for a specific amount of larvae and (g) single larval weight (g) are reported.
